# Biochemical and Anatomical Changes and Yield Reduction in Rice (*Oryza sativa *L.) under Varied Salinity Regimes

**DOI:** 10.1155/2014/208584

**Published:** 2014-01-22

**Authors:** M. A. Hakim, Abdul Shukor Juraimi, M. M. Hanafi, Mohd Razi Ismail, Ahmad Selamat, M. Y. Rafii, M. A. Latif

**Affiliations:** ^1^Department of Land Management, University Putra Malaysia (UPM), Serdang, 43400 Selangor, Malaysia; ^2^Department of Agricultural Chemistry, Hajee Mohammad Danesh and Technology University, Dinajpur 5200, Bangladesh; ^3^Department of Crop Science, Faculty of Agriculture, University Putra Malaysia (UPM), Serdang, 43400 Selangor, Malaysia; ^4^Institute of Tropical Agriculture, University Putra Malaysia (UPM), Serdang, 43400 Selangor, Malaysia; ^5^Bangladesh Rice Research Institute, Gazipur 1701, Bangladesh

## Abstract

Five Malaysian rice (*Oryza sativa* L.) varieties, MR33, MR52, MR211, MR219, and MR232, were tested in pot culture under different salinity regimes for biochemical response, physiological activity, and grain yield. Three different levels of salt stresses, namely, 4, 8, and 12 dS m^−1^, were used in a randomized complete block design with four replications under glass house conditions. The results revealed that the chlorophyll content, proline, sugar content, soluble protein, free amino acid, and yield per plant of all the genotypes were influenced by different salinity levels. The chlorophyll content was observed to decrease with salinity level but the proline increased with salinity levels in all varieties. Reducing sugar and total sugar increased up to 8 dS m^−1^ and decreased up to 12 dS m^−1^. Nonreducing sugar decreased with increasing the salinity levels in all varieties. Soluble protein and free amino acid also decreased with increasing salinity levels. Cortical cells of MR211 and MR232 did not show cell collapse up to 8 dS m^−1^ salinity levels compared to susceptible checks (IR20 and BRRI dhan29). Therefore, considering all parameters, MR211 and MR232 showed better salinity tolerance among the tested varieties. Both cluster and principal component analyses depict the similar results.

## 1. Introduction

Crops are often exposed to salinity immediately after planting in saline soil or in areas inundated by sea water or irrigated with brackish water. Salinity is a problem over vast areas in the South and South-East Asia [1]. Salinity is a major abiotic stress to rice production at all growth stages [2]. Up to fifty percent yield loss may occur in salinity-sensitive rice varieties [3]. Peel et al. [4] found that salt suppresses plant growth at low concentrations, and at higher it concentrations can cause plant mortality. The major inhibitory effect of salinity on plant growth has been attributed to osmotic effects, ion toxicity, and nutritional imbalance leading to reduction in photosynthetic activities and other physiological disorders [5]. The biosynthesis of photosynthetic pigments is also affected with increasing salinity stress [5]. The proline content and other biochemical constituents such as soluble carbohydrates and proteins are also influenced significantly with increasing salt levels [6]. The compatible osmolytes found in higher plants are of low molecular weight sugars, organic acids, amino acids, proteins, and quaternary ammonium compounds. The accumulation of soluble carbohydrates in plants has been widely reported as response to salinity, despite a significant decrease in net CO2 assimilation rate [7]. Amino acids have been reported to have accumulated in higher plants under salinity stress [8].

In Malaysia, rice is the third top raking crop, mainly grown in eight granaries covering an area of 205,548 ha in Peninsular Malaysia [9] but meeting about 70% of the local demand [10]. To fulfill the current domestic and increasing future needs, Malaysia must expand its rice area [3]. It is assumed that the salinity problem would affect 100,000 ha of rice area by 2056 [3]. Continuous intrusion of saline water would result in dwindling of rice area leading to food shortages in domestic and global markets. The increasing population makes its mandatory for more research and technological advancement to increase rice production for consumption within the nation [11]. Nevertheless, researchers and policy makers must pave ways for the efficient exploitation of the salinity prone areas. The assortment of salt-tolerant rice cultivar(s) might be the finest approach to bring the saline areas under rice culture [5, 12]. By now, much work have been done to comprehend the influences of saline habitats on seed germination, growth, reproduction, and population dynamics of crop plants [13]. But information on Malaysian rice and its saline zones is scanty. Selection of salt-tolerant rice varieties is thus very important to mitigate salinity in coastal regions. This work was, therefore, executed as an in-depth search to explore the leeway of developing salt-tolerant rice cultivars as well as fruitful rice production on the saline soils in Malaysia. The study was, therefore, designed as an attempt to characterize the influence of salt stresses on the basis of biochemical, anatomical, and yield of Malaysian rice and to select salinity tolerant rice varieties for coastal areas of Peninsular Malaysia.

## 2. Materials and Methods

### 2.1. Experimental Site, Design, and Soil Properties

The study was conducted in pots (33 cm diameter × 23 cm depth) at the glasshouse of Universiti Putra Malaysia (3°00′ 21.34′′ N, 101°42′ 15.06′′ E, 37 m elevation) during the period from October 2010 to January 2011. The experiment was organized in a randomized complete block design (RCBD) with four replications. Soil for this experiment was collected from farmer's rice field in Tanjung Karang. The experimental soil was loamy clay in texture (18.3% sand, 43.7% silt, 38% clay) and acidic in reaction (pH 6.1) with 1.02% organic carbon, EC-1.56 dS m^−1^; soil nutrient status was 0.19% total N, 11.12 ppm available P, 122 ppm available K, 620 ppm Ca, 290 ppm, 7.63 ppm S, and 0.96 ppm Zn.

### 2.2. Plant Material

Eight rice varieties were chosen, five were of Malaysian origin (namely, MR33, MR52, MR211, MR232, and MR219) and three were of exotic origin (BRRI dhan29, IR20, and Pokkali). BRRI dhan 29 and IR20 were salt sensitive and used as negative control. On the other hand, Pokkali is a well-known salt resistant cultivar and was used as a positive control.

### 2.3. Treatments

Four salinity levels were employed, namely, 0, 4, 8 and 12 dS m^−1^ in this study. Different salinity levels were developed by dissolving commercial salt (NaCl, Batch# 088K0089, Sigma-Aldrich Co., USA) at the rate of 2.54 g per litre distilled water for 4 dS m^−1^ salinity level, 5.08 g per litre distilled water for 8 dS m^−1^ salinity level, and 7.62 g per litre distilled water for 12 dS m^−1^ salinity level. Distilled water was used as the control, that is, 0 salinity. Different salinity levels were reconfirmed by electrical conductivity meter (model: Z 865/SCHOTT Instruments, Germany), and necessary adjustment was made.

### 2.4. Methodology

Every pot was filled with 10 kg soil well mixed with urea, triple supper phosphate (TSP), muriate of potash (MOP), and gypsum as sources of N, P, K, and S at the rate of 60 kg N, 80 kg P_2_O_5_, 150 kg K_2_O, and 20 kg S ha^−1^, respectively. Three weeks old rice seedlings were transplanted into the pots with three seedlings per pot. Two weeks after transplanting, salt treatments were applied. To avoid osmotic shock, salt solutions were added in three equal splits on alternate days until the expected conductivity (0, 4, 8, and 12 dS m^−1^) was reached. Urea was top dressed twice at 30 and 60 days after transplanting at 60 kg N/ha. Standard agronomic practices were adopted and crop protection measures were carried out as necessary [14]. Leachates of salt solutions were collected daily from each pot and monitored for electrical conductivity (EC), and adjustments were made when necessary. Conductivity of soil was determined using conductivity meter (Model: ECTestr, Spectrum Technologies, Inc.). The crop was harvested at full maturity (when 90% grains became golden yellow), and the grain weight recorded. The yield was adjusted at 12% moisture basis.

### 2.5. Determination of Biochemical Parameters

Chlorophyll content such as chlorophyll-a, chlorophyll-b, and total chlorophyll were determined from 45 days aged leaf samples using the method of Coombs et al. [15]. Proline content was estimated according to the method of Bates et al. [16].

Total soluble sugar content was determined according to the procedure outlined by Smogyi [17]. Reducing sugar content was determined by Somogyi-Nelson method [18]. Soluble protein content in leaf samples was determined according to the method of Lowry et al. [19]. Extraction and estimation of total free amino acid following the procedure outlined by Yemm and Cocking [20].

### 2.6. Root Histology Using Scanning Electron Microscopy

Root histology was observed using a Scanning Electron Microscope (SEM) (JEOL JSM-5610LV, Japan). Roots of four varieties were collected at 60 days after transplanting and sampled at two root zones (root tipsat 0–0.5 cm from the tip, and mature roots). Roots were cut to length of 5 mm with a sharp blade. The excised roots were placed in formalin acetic acid (FAA) and vacuumed for 1 hour at 650 mm Hg. Specimens were postfixed in 1% osmium tetroxide for 2 h, dehydrated for 30 min in each graded ethanol series of 30, 50, 70, 90, 95, and 100%, and dried in Baltec CPD 030 critical point dryer apparatus. The tissues were mounted on stubs, coated with gold using Auto Fine Coater (JEOL JFC-1600, Japan) for 20 min, and viewed under Scanning Electron Microscope (JEOL JSM-5610LV, Japan).

### 2.7. Statistical Analysis

All data were analyzed by analysis of variance procedure (ANOVA), and means were separated by least significant difference (LSD) at the 5% probability level using Statistical Analysis System software (SAS, version 9.0). Regression analysis was performed with mean value to determine the relationship among variables and salinity levels. Data were analyzed using Euclidian distance and run on PAST multivariate software. This distance matrix was used to produce a dendrogram for clustering and depicting of the genetic relationships. These data sets were then subjected to PCA and variances for different components were determined.

## 3. Results 

### 3.1. Chlorophyll Content

Chlorophyll-a in the eight rice varieties was significantly affected by salinity (Table 2). The highest chlorophyll-a was observed in IR20 (10.49), while the lowest was observed in MR219 at 12 dS m^−1^ (3.81). At 4 dS m^−1^, the least affected varieties were MR33, MR211, and MR232 having more than 95% chlorophyll-a compared to control. The most affected variety was IR20 with 81% chlorophyll-a related to the control. At 8 dS m^−1^, MR211 produced maximum chlorophyll-a with 87%, and the lowest was obtained from IR20 (59% relative to the control). However, at 12 dS m^−1^, all varieties were more influenced by salt stress, but the highest amount of chlorophyll-a was recorded in MR232 followed by Pokkali and MR211 with more than 60% relative to the control. However, the lowest chlorophyll-a was obtained in MR219 followed by IR20 and BRRI dhan29 with less than 40% relative to the control.

The effect of salinity levels on chlorophyll-b was significant (Table 3). The highest chlorophyll-b content (5.85 mg cm^−2^) was recorded in IR20, while the lowest chlorophyll-b content (1.25 mg cm^−2^) was observed in MR219 at 12 dS m^−1^. At 4 dS m^−1^, the highest amount of chlorophyll-b content was noted in MR232, followed by MR211 with relative values of ≥80%, while the lowest amount was recorded in MR52 with 66% relative to the control. At 8 dS m^−1^, a higher chlorophyll-b content was obtained in MR232 (60%), and the lowest value was found in MR219 (38%). A similar trend was observed at the higher salinity level of 12 dS m^−1^.

**Figure 1:**
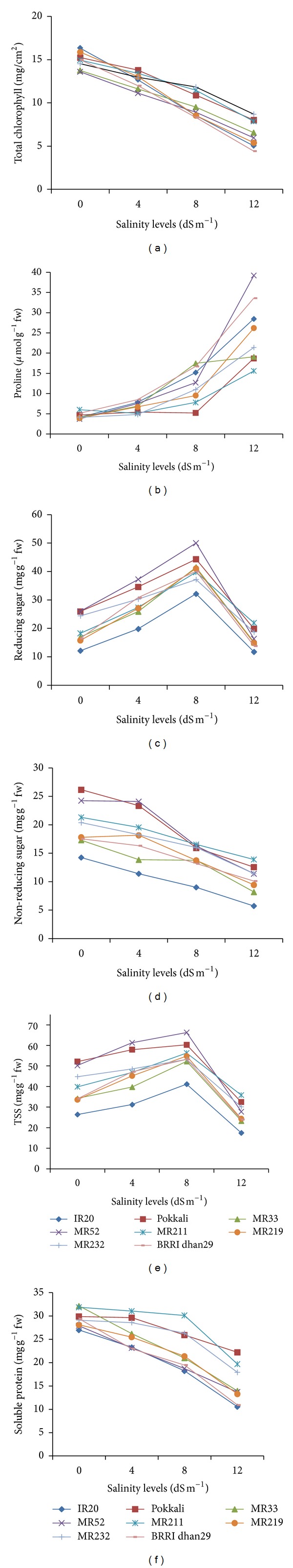
Effect of salinity on total chlorophyll content, proline content, reducing sugar, nonreducing sugar, total soluble sugar, and soluble protein of eight rice varieties.

Total chlorophyll content decreased with increasing of salt stress in all rice varieties (Figure 1(a)). At 4 dS m^−1^, Pokkali and MR211 consisted of comparatively higher amounts of total chlorophyll. MR211, Pokkali, and M232 showed lesser reductions, while severe reductions were observed in IR20, BRRI dhan29, and MR219 due to salt stresses at 8 and 12 dS m^−1^. The chlorophyll a/b ratio varied significantly with salinity levels (Table 1), but there was no specific trend. The results, however, clearly indicated that chlorophyll content was significantly influenced by increasing salinity.

### 3.2. Proline Content

The accumulation of proline was significantly influenced by salinity. The proline content increased with increasing of salinity levels in all varieties (Figure 1(b)). At 4 dS m^−1^, there was a slight increase in proline content in all varieties, except MR211. At 8 dS m^−1^, the highest proline accumulation was found in MR33, while the lowest accumulation was in Pokkali. The accumulation increased sharply in all varieties at 12 dS m^−1^, but the highest increment (39.2 *μ*mol g^−1^ fw) was recorded in MR52, while the lowest (12.2 *μ*mol g^−1^ fw) was in MR211.

### 3.3. Sugar Content

The results showed that salinity levels significantly influenced the content of reducing sugars in rice leaves (Table 1). The reducing sugars increased with increasing of salinity levels in all varieties up to 8 dS m^−1^ level, and after it decreased considerably. At 4 dS m^−1^, maximum reducing sugar was found in MR52 (37.22 mg g^−1^ fw) followed by Pokkali (34.57 mg g^−1^ fw), while the minimum amount was observed in IR20 (19.82 mg g^−1^ fw). A similar trend was observed at 8 dS m^−1^. However, at 12 dS m^−1^, the reducing sugar in leaves decreased with increasing salinity in all varieties and the highest value was observed in MR211 (21.92 mg g^−1^ fw), while the lowest (13.81 mg g^−1^ fw) was recorded in BRRI dhan29 (Figure 1(c)). The reducing sugar followed a polynomial response (*R* ^2^ = 0.8001) with increasing salinity (Figure 3).

The main effect of salinity on nonreducing sugar was found to be significant (Table 1). At 4 dS m^−1^, the highest amount of nonreducing sugar was recorded in MR52 and the lowest was obtained in IR20. At 8 dS m^−1^, the nonreducing sugars decreased in all varieties compared to the control. The highest value was observed in MR211 (16.54 mg g^−1^ fw) while the lowest was recorded in IR20 (9.0 mg g^−1^ fw). Similar response was observed at the higher salinity level (Figure 1(d)). The nonreducing sugar decreased linearly (*R* ^2^ = 0.9765) with increasing salinity levels (Figure 3).

The effect of salinity on total sugar contents was significant (Table 1).The interaction effects of total soluble sugar are presented in Figure 1(e) At 4 dS m^−1^, the content of total sugars was the highest (61.29 mg g^−1^ fw) in MR52 and the lowest (31.22 mg g^−1^ fw) was in IR20. A similar trend was observed at 8 dS m^−1^. However, at 12 dS m^−1^, the total sugar content significantly decreased in all varieties. At this salinity level, the highest value recorded was in MR211, while the lowest value was in IR20. The total soluble sugar showed a polynomial (*R* ^2^ = 0.8568) response to the effect of salinity (Figure 3).

### 3.4. Free Amino Acid

The content of free amino acid in rice leaves of eight rice varieties significantly decreased with increasing of salinity levels (Table 1). The application of different levels of salinity decreased the accumulation of free amino acid in leaves of all rice varieties and the reduction of free amino acid was prominent in salt-sensitive varieties (IR20 and BRRI dhan 29).

The results presented in Table 1 showed that the content of free amino acid in the eight rice varieties varied significantly due to the mean effect of salinity levels. The highest amount of free amino acid (16.31 mg g^−1^ fw) was obtained in the leaves of MR33, which was statistically identical with MR211 while the lowest was (7.87 mg g^−1^ fw) in IR20.

**Figure 2:**
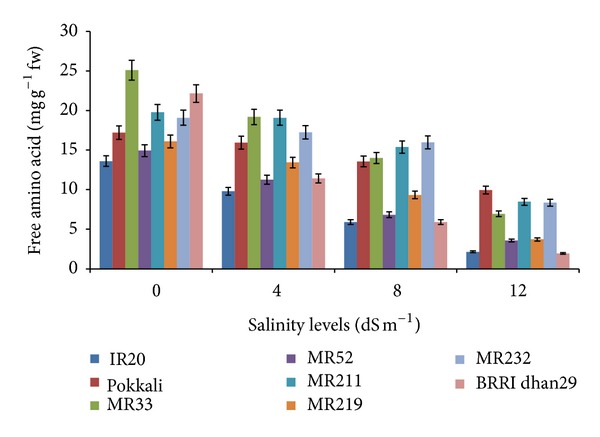
Effect of salinity on free amino acid of eight rice varieties.

**Figure 3:**
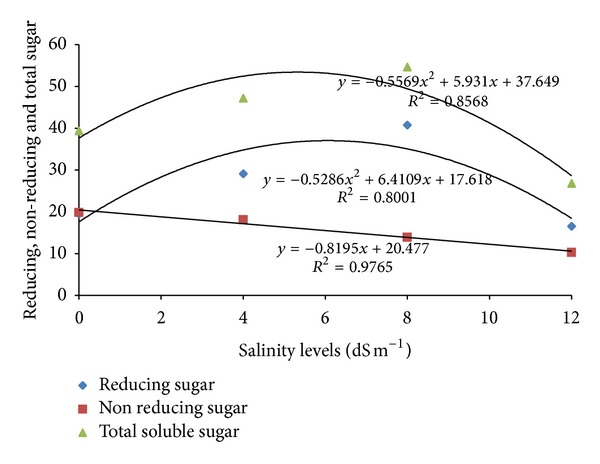
Salinity responses in reducing, nonreducing, and total sugars in rice leaves.

The different salinity levels had significant effect on free amino acid content in leaves of eight rice varieties (Figure 2). At 4 dS m^−1^, the highest amount of free amino acids (19.20 mg g^−1^ fw) was observed in MR33 followed by MR211 (19.09 mg g^−1^ fw). The lowest amount (9.81 mg g^−1^ fw) was found in the IR20. At 8 dS m^−1^ level of salinity, the highest amount of free amino acids was produced in MR232 (15.98 mg g^−1^ fw) and the lowest was recorded in BRRI dhan29 (5.90 mg g^−1^ fw). It was observed that the highest value in Pokkali followed by MR211 and MR232 at higher salinity level. Though the leaves of rice variety BRRI dhan29 contained 2nd highest amount of free amino acids at control treatment but it reduced dramatically with increasing the salinity level (Figure 2).

### 3.5. Soluble Protein

The effect of salinity on soluble protein content in rice leaves was significant (Table 1). The concentration of soluble protein in rice leaves decreased at higher salinity level. At 4 dS m^−1^, the highest soluble protein content (31.08 mg g^−1^ fw) was found in MR211, while the lowest (22.99 mg g^−1^ fw) was obtained in BRRI dhan29. At 8 dS m^−1^, higher soluble protein content (30.12 mg g^−1^ fw) was observed in MR211, followed by Pokkali and MR232, while the lowest (18.23 mg g^−1^ fw) values were observed in IR20, followed by BRRI dhan29 and MR52. However, at higher salinity levels, the protein contents decreased more in all varieties, but the highest decrease was in IR20, followed by BRRI dhan29, while the least reduction was in Pokkali, followed by MR211 and MR232 (Figure 1(f)). Protein content is an important indicator of physiological status of plants.

### 3.6. Rice Grain Yield

A significant reduction in overall grain yield (g hill^−1^) was observed in all varieties at different level of salinity. Grain yield of MR211, MR232, and Pokkali varieties was reduced by 10–14%, 38–45%, and 72–75% at 4, 8, and 12 dS m^−1^ of salinity, respectively, demonstrating them as the salt-tolerant varieties among the studied population of 8 rice varieties (Table 4). At 4 dS m^−1^, the maximum yield reduction was observed in IR20 (64%) and BRRI dhan29 (41%), respectively. The grain yield in these varieties was totally unobtainable at 8 and 12 dS m^−1^ of salinity, reflecting them as the most sensitive varieties to salinity. MR219, MR52, and MR33 demonstrated medium level of sensitivity. These varieties lost 26–53%, 69–78%, and 100% grain yield from the control group at 4, 8, and 12 dS m^−1^ of salinity, respectively.

### 3.7. Effect of Salinity on Root Histology

**Figure 4:**
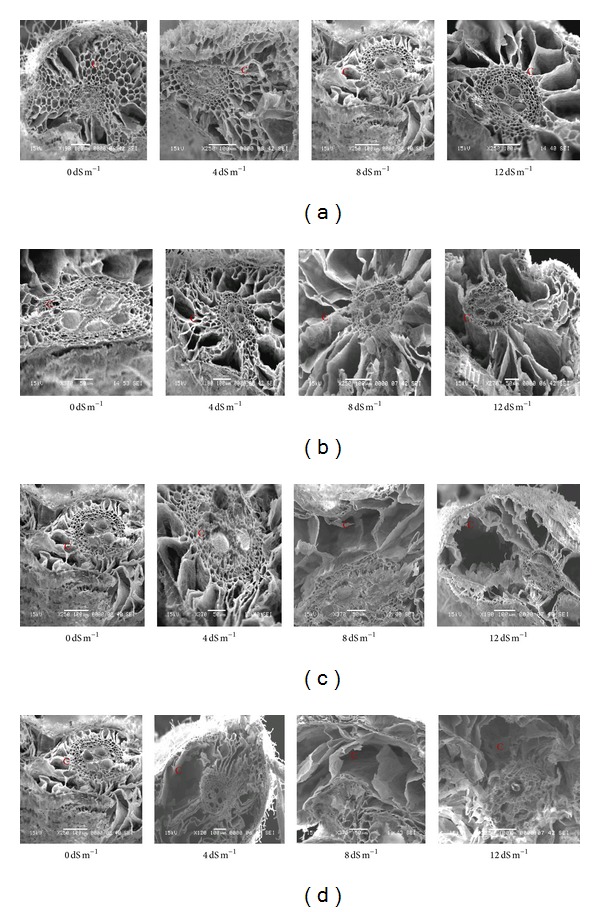
Scanning electron micrographs showing salinity effects on root cortical tissue of (a) MR232, (b) MR211, (c) IR20, and (d) BRRI dhan 29.

The cell damage in the root cortex due to salinity treatments was attributed to root cell collapse. Cortical cells of MR211 and MR232 did not show cell collapse in 0, 4, and 8 dS m^−1^ salinity treatments (Figure 4). MR33 did not show cortical cell collapse at 0 and 4 dS m^−1^, but for IR20 showed some cell collapse in the 8 dS m^−1^ salinity treatment (Figure 4). Some cortical cells of IR20 and BRRI dhan29 showed some collapse in 4 dS m^−1^, and this condition became severe at the higher salinity treatment (8 dS m^−1^and 12 dS m^−1^). In comparison with susceptible varieties of IR20 and BRRI dhan29, the cortical cells of MR211 and MR232 varieties showed good condition up to 12 dS m^−1^.

### 3.8. Cluster Analysis

**Figure 5:**
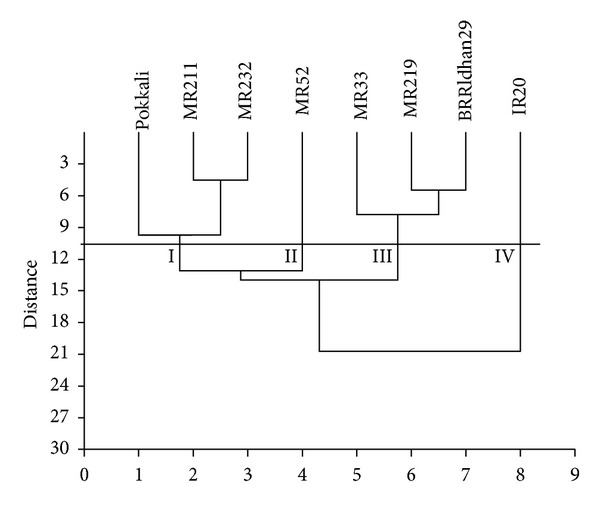
A UPGMA dendrogram of 11 biochemical traits derived from 8 salinity tolerant and susceptible genotypes of rice.

The biochemical and yield data were used to calculate the Euclidean distances between the genotypes of salinity tolerant and susceptible and an UPGMA dendrogram was constructed (Figure 5). In this dendrogram, 8 genotypes were appeared to form four major clusters at distance level 10.5. Clusters I, II, III, and IV had three (Pokkali, MR211, and MR232), one (MR52), three (MR33, MR219, and BRRI dhan29), and one (IR20) members, respectively. Cluster analysis clearly stated that salinity tolerant varieties (Pokkali, MR211, and MR232) grouped into one cluster while other moderate or susceptible varieties formed into different clusters, II, III, and IV.

### 3.9. Principal Component Analysis (PCA)

**Figure 6:**
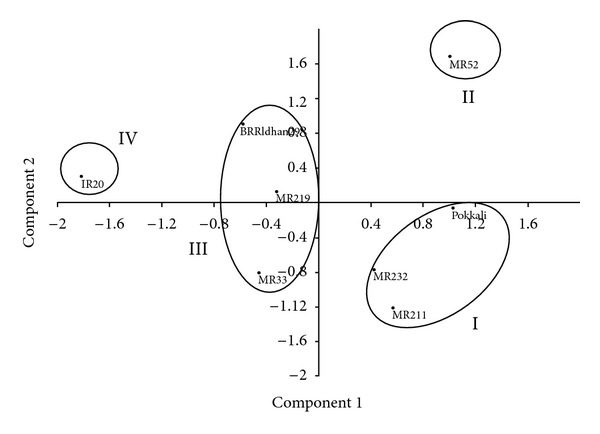
A PCA two-dimensional graph indicating variations among 8 salinity tolerant and susceptible genotypes of rice.

In order to assess the patterns of variation, PCA was done by considering all the 11 characters. The first three components of PCA were explained by 97.2% of the total variation (Figure 6). Only the first component which was accounted for 74.2% of the total variation which was attributed to proline, reducing sugar, nonreducing sugar, total soluble sugar, free amino acid, total soluble protein, and grain yield. In PCA two-dimensional graph, salinity tolerant genotypes, Pokkali, MR232, and MR211 were grouped together and was away from other clusters (Figure 6). Groups in two-dimensional graph in PCA were similar to the groups of cluster analysis (Figure 5), that is, both analyses corroborated each other.

## 4. Discussion

Soil salinity, one of the most serious problems on planting areas, has the most obstructive impact on crop production in the world. This crisis attracts many scientists to work towards overcoming this obstruction by improving salt-tolerant lines. Indica rice is an important crop in the world, with its subspecies distributed in several countries. Presently, the production and planting area of rice are greatly menaced by soil salinity.

In this study chlorophyll-a, chlorophyll-b, and total chlorophyll were significantly decreased under saline condition. The higher amount of chlorophyll was observed in MR211 and MR232 whereas the other varieties showed significantly lower chlorophyll contents. The discrepancy of these results might be due to differences in the rice varieties. Reduction in chlorophyll concentrations is probably due to loss of photosynthetic capacity and the inhibitory effect of the accumulated ions on the biosynthesis of the chlorophyll fractions. Chlorophyll degradation is induced by many stresses, leading to changes of certain enzyme activities, photosynthetic electron transport, carbon metabolism, and photophosphorylation in photosynthesis. During salt stress, salt-sensitive plants clearly showed chlorophyll degradation and growth reduction. Salt-sensitive rice generally had lower chlorophyll contents than salt-tolerant rice cultivars [21]. Mitsuya et al. [22] suggested that decrease in chlorophyll content was caused by a light-dependent reaction and not directly by accumulation of excess salt. The chlorophyll pigments in rice are sensitive to salt stress especially in salt susceptible varieties [5, 23], and chlorophyll-b was more sensitive than chlorophyll-a [6, 24]. These results are in agreement with the present study where chlorophyll pigments in rice leaves were influenced significantly under salinity stress.

Accumulation of proline in the cytoplasm is accompanied by a reduction in the concentrations of less compatible solutes, for example, K^+^ and glutamate, and an increase in cytosolic water volume. It has been reported that the accumulation of proline occurred up to 3 days after treatment with 200 mM NaCl in tobacco10 and up to 10 days with 100 mM NaCl in rice. This study showed that the accumulation of proline occurred in increasing pattern with increasing salinity levels. Proline may play a role at protecting chlorophyll, a photosynthetic pigment of the chloroplast. The accumulation of compatible solutes such as proline is an important mechanism in higher plants under salt stress. Proline accumulation in salt-stressed plants is a primary defence response to maintain osmotic pressure in a cell. Many researchers have reported the significant role of proline in osmotic adjustment and protection of cell structure in many crops [25]. Chutipaijit et al. [26] reported that free proline content of rice varieties was significantly increased with increasing salinity levels. Wanichananan et al. [27] found that the proline content of rice seedlings was affected by the presence of NaCl in the growth medium, and the proline content positively correlated with the NaCl. Similar result was observed by Moradi and Ismail [1] where proline concentration increased significantly in all three rice lines with increasing salinity levels.

Zahra et al. [28] also reported that sugar levels in rice leaves increased significantly under salt stress. However, Alamgir and Ali [29] observed that salinity reduced sugar content in four varieties but increased sugar content in five other varieties. Ruan et al. [30] on the other hand reported their findings on total soluble sugars in hybrid rice under salt stress (50, 100, and 150 mM NaCl concentrations) and observed that the trends were not regular.

Our result is supported by Razzaque et al. [21] who found that soluble protein content in leaves of rice genotypes increased significantly with increase in salinity levels, this increasing pattern continued up to 9 dS m^−1^ and decreased thereafter. Kumar et al. [31] found that protein content of some indica rice genotypes increased up to 100 mM NaCl concentration and decreased thereafter with increasing salinity levels. Sultana et al. [32] observed that protein of rice was decreased with increasing the salinity levels. Amirjani [6] observed that the soluble protein contents in rice seedlings were significantly influenced with increasing salinity and the total protein content decreased at higher salinity levels (200 mM). Total soluble protein contents of tomato cultivars were significantly decreased with increasing salinity levels but some varieties appeared initially increased [24]. Demiral and Türkan [33] reported an increase in soluble protein of Pokkali and a decrease in the soluble protein of IR-28 under salt stress.

In this study, grain yield loss occurred due to effect of salinity in all varieties but yield performances of MR211 and MR232 were better in all salinity levels than the other varieties in comparison with salt-tolerant check Pokkali. Probable cause for lower grain yield in susceptible varieties was reduction in cell metabolic activities which limit the cell wall elasticity, and thus cell walls become rigid and consequently the turgor pressure of cell decreases. The other possible causes could be the shrinkage of cell contents, reduced development and differentiation of tissues, imbalanced nutrition, damage of membranes, and disturbed avoidance mechanisms [5, 34]. The grain yield plant^−1^ of rice genotypes was significantly reduced under salinity stress [34]. Similar result was also found by Mahmood et al. [35] where rice grain yield of rice was significantly decreased with increasing salinity levels.

Based on 11 characters including biochemical and yield, the 8 genotypes of salinity tolerant and susceptible grouped into four clusters which indicate a high level of variation in the genotypes. The existence of biochemical variation among genotypes was further substantiated by principal component analysis. Both cluster and principal component analyses clearly demonstrated that MR211 and MR233 were tolerant to salinity and within salinity tolerant varieties biochemical variations were low. These varieties could be used in saline prone areas in Malaysia. Several authors also used more than one multivariate analysis to identify the desired genotypes [36, 37].

## 5. Conclusion

The present study revealed that the varieties tested in the study, MR211 and MR232 were found to be least affected, followed by MR33, and MR52 due to salinity. Based on the overall results on biochemical, anatomical, and yield performances, it is concluded that varieties MR232 and MR211 were comparatively salinity tolerant, while MR33 and MR52 were observed to be moderately tolerant, and IR20 and MR219 were susceptible varieties.

## Figures and Tables

**Table 1:** The main effect of salinity on biochemical characteristics and yield of eight rice varieties.

Salinity levels (dSm^−1^)	Chlorophyll-a (mg/cm^2^)	Chlorophyll-b (mg/cm^2^)	Total chlorophyll (mg/cm^2^)	Chlorophyll a/b	Proline (*μ*mol g^−1^ fw)	Reducing sugar (mg g^−1^ fw)	Nonreducing sugar (mg g^−1^ fw)	Total soluble sugar (mg g^−1^ fw)	Free amino acid (mg g^−1^ fw)	Total soluble protein (mg g^−1^ fw)	Grain yield (g hill^−1^)
0	8.99^a^	4.67^a^	13.67^a^	1.93^c^	4.64^d^	19.50^c^	19.87^a^	39.38^c^	18.50^a^	29.36^a^	17.78^a^
4	8.11^b^	3.60^b^	11.71^b^	2.28^b^	5.71^c^	29.13^b^	18.11^b^	47.24^b^	14.6^b^	26.30^b^	11.63^b^
8	6.59^c^	2.57^c^	9.15^c^	2.57^a^	11.33^b^	40.75^a^	13.92^c^	54.68^a^	10.86^c^	22.64^c^	5.73^c^
12	4.14^d^	1.71^d^	5.86^d^	2.46^ab^	23.75^a^	16.54^d^	10.34^d^	26.89^d^	5.64^d^	15.28^d^	1.65^d^
*Variety *											
IR20	6.91^bc^	3.16^b^	10.07^bc^	2.36^abc^	13.9^bc^	18.96^e^	10.09^f^	29.09^d^	7.87^e^	19.77^e^	4.97^d^
Pokkali	7.39^ab^	3.62^a^	11.01^a^	2.10^c^	8.58^e^	31.18^a^	19.50^a^	50.58^a^	14.17^b^	26.79^a^	7.64^c^
MR33	6.72^cd^	2.72^c^	9.44^de^	2.58^a^	11.69^cd^	24.85^d^	12.52^e^	37.38^c^	16.31^a^	23.63^bc^	9.09^b^
MR52	6.22^d^	2.93^c^	9.19^e^	2.16^bc^	15.37^b^	32.39^a^	19.0^ab^	51.39^a^	9.15^de^	20.86^de^	9.10^b^
MR211	7.70^a^	3.29^b^	10.99^a^	2.39^ab^	8.625^e^	26.83^bc^	17.82^bc^	44.65^b^	15.68^a^	28.19^a^	12.41^a^
MR219	6.95^bc^	2.96^c^	9.91^cd^	2.45^a^	11.57^cd^	24.71^d^	14.77^d^	39.48	10.65^c^	22.06^cd^	8.79^b^
MR232	7.39^ab^	3.20^b^	10.60^ab^	2.32^abc^	10.325^d^	27.64^b^	16.50^c^	44.15^b^	15.17^ab^	24.49^b^	13.49^a^
BRRI dhan29	6.38^d^	3.22^b^	9.61^cde^	2.14^bc^	15.97^a^	25.29^cd^	14.29^d^	39.58^c^	10.36^cd^	20.70^de^	5.98^d^

*F*-test	∗∗	∗∗	∗∗	∗∗	∗∗	∗∗	∗∗	∗∗	∗∗	∗∗	∗∗
CV (%)	9.18	7.25	6.73	13.86	11.47	9.49	14.64	8.17	15.21	9.79	16.37

Means with the same letter in the columns do not differ significantly (*P* ≤ 0.05), **significant in 1% level.

**Table 2:** Effect of salinity on chlorophyll-a content (mg cm^−2^) of eight rice varieties.

Varieties	Salinity levels (dS m^−1^)			
	0	4	8	12
IR20	10.49^a^ (100)	8.53^abc^ (81)	6.24^c^ (59)	3.85^d^ (37)
Pokkali	9.78^abc^ (100)	9.11^ab^ (93)	7.87^a^ (77)	5.93^a^ (61)
MR33	8.85^bc^ (100)	8.59^abc^ (97)	7.08^b^ (80)	5.04^b^ (57)
MR52	8.26^c^ (100)	7.28^b^ (89)	6.01^c^ (73)	4.05^bc^ (49)
MR211	9.85^ab^ (100)	9.44^a^ (96)	8.52^a^ (87)	5.96^a^ (61)
MR219	10.28^ab^ (100)	8.98^abc^ (88)	6.41^bc^ (62)	3.81^d^ (36)
MR232	9.64^ab^ (100)	9.24^a^ (96)	7.95^ab^ (83)	6.12^a^ (64)
BRRI dhan29	9.55^abc^ (100)	7.54^b^ (79)	6.0^c^ (63)	3.73^d^ (39)

Means within columns with the same letters are not significantly different (LSD, *P* ≤ 0.05).

Values within parenthesis indicate percent relative to the control.

**Table 3:** Effect of salinity on chlorophyll-b content (mg cm^−2^) of eight rice varieties.

Varieties	Salinity levels (dS m^−1^)			
	0	4	8	12
IR20	5.85^a^ (100)	4.15^ab^ (71)	2.30^cd^ (39)	1.37^c^ (23)
Pokkali	5.80^a^ (100)	4.65^a^ (81)	3.02^a^ (52)	2.07^a^ (36)
MR33	4.88^c^ (100)	3.44^cd^ (71)	2.42^cd^ (50)	1.52^bc^ (31)
MR52	4.83^c^ (100)	3.19^d^ (66)	2.61^bc^ (54)	1.68^b^ (35)
MR211	5.10^bc^ (100)	4.18^ab^ (82)	2.92^ab^ (57)	2.09^a^ (41)
MR219	5.61^ab^ (100)	4.04^ab^ (72)	2.15^d^ (38)	1.25^c^ (22)
MR232	4.90^c^ (100)	4.13^ab^ (85)	2.95^ab^ (60)	2.07^a^ (42)
BRRI dhan29	5.66^a^ (100)	4.00^bc^ (71)	2.23^d^ (40)	1.27^c^ (23)

Means within columns with the same letters are not significantly different (LSD, *P* ≤ 0.05).

Values within parenthesis indicate percent relative to the control.

**Table 4:** Effect of salinity on the grain yield (g hill^−1^) of eight rice varieties.

Rice variety	Salinity levels (dS m^−1^)			
	0 (control)	4	8	12
IR20	14.65^cd^	5.24^d^ (36)	0.0^e^	0.0^c^
Pokkali	11.93^d^	10.29^c^ (86)	7.05^c^ (60)	3.31^b^ (28)
MR33	19.44^ab^	12.37^bc^ (64)	5.64^cd^ (29)	0.0^c^
MR52	18.59^ab^	13.36^b^ (72)	5.73^cd^ (31)	0.0^c^
MR211	18.85^ab^	17.00^a^ (90)	10.36^b^ (55)	4.83^a^ (26)
MR219	21.48^a^	10.00^c^ (47)	4.67^d^ (22)	0.0^c^
MR232	20.10^ab^	18.07^a^ (90)	12.42^a^ (62)	5.08^a^ (25)
BRRI dhan29	17.26^bc^	6.72^d^ (59)	0.0^e^	0.0^c^

Means with the same letter in the columns do not differ significantly (*P* ≤ 0.05).

Values within parenthesis indicate percent relative to the control.
